# Ultraviolet Radiation-Induced Tolerogenic Dendritic Cells in Skin: Insights and Mechanisms

**DOI:** 10.3390/cells14040308

**Published:** 2025-02-18

**Authors:** Gelare Ghajar-Rahimi, Nabiha Yusuf, Hui Xu

**Affiliations:** Department of Dermatology, Heersink School of Medicine, University of Alabama, Birmingham, AL 35294, USA

**Keywords:** ultraviolet radiation, skin, tolerogenic dendritic cells, immunology, immunosuppression

## Abstract

Ultraviolet (UV) radiation has profound effects on the immune system, including the induction of tolerogenic dendritic cells (DCs), which contribute to immune suppression and tolerance. This review explores the roles of conventional CD11c⁺ DCs, as well as cutaneous Langerhans cells and CD11b⁺ myeloid cells, in UV-induced immune modulation. Two key mechanisms underlying the immunosuppressive relationship between UV and DCs are discussed: the inactivation of DCs and the induction of tolerogenic DCs. DCs serve as a critical link between the innate and adaptive immune systems, serving as professional antigen-presenting cells. In this context, we explore how UV-induced DCs influence the activity of specific T cell subsets, including regulatory T lymphocytes and T helper cells, and shape immune outcomes. Finally, we highlight the implications of UV-induced tolerogenic DCs in select dermatologic pathologies, including cutaneous lupus, polymorphic light eruption, and skin cancer. Understanding the mechanisms by which UV radiation alters DC function offers insights into the complex interplay between environmental factors and immune regulation, providing potential avenues for preventive and therapeutic intervention in UV-induced skin diseases.

## 1. Introduction

Ultraviolet (UV) radiation, a ubiquitous environmental factor, profoundly influences the immune system, particularly within the skin, where it induces a state of immunosuppression. Among the cellular mediators of this immunomodulatory effect, dendritic cells (DCs)—the sentinel antigen-presenting cells (APCs) of the immune system—play a pivotal role. UV-induced changes in DC function can impair T cell function, promote immune tolerance, and reshape the skin’s immune landscape.

This review focuses on the mechanisms by which UV radiation influences the tolerogenic capacity of DCs in the skin. We explore the roles of various DC subsets, including Langerhans cells (LCs), conventional CD11c^+^ cells, and CD11b^+^ myeloid cells, highlighting their contributions to UV-induced immunosuppression. We also discuss the molecular pathways implicated in the modulation of DC activity, including DNA damage, cytokine signaling, and apoptotic processes. Furthermore, we examine the clinical implications of these findings, highlighting the role of UV-induced DCs in the development of conditions such as cutaneous lupus erythematosus, polymorphic light eruption, and skin cancers. By synthesizing recent advances in the field, this review aims to provide a comprehensive understanding of UV-induced tolerogenic DCs and their broader immunological and clinical significance.

## 2. Various Cutaneous Immune Cells Implicated in UV-Induced Immune Suppression

This review discusses three broad categories of dendritic cells, reflecting the prevailing trends in the current literature. While the techniques and markers used to define these categories vary somewhat, a summary is provided in Table 1. It is important to note that the classification of immune cells is continually evolving as advancements in molecular techniques, multiparameter imaging, flow cytometry, and transcriptomics drive more specific characterization.

### 2.1. Langerhans Cells

LCs, first described by Paul Langerhans in 1868, were the earliest cells to be identified as DCs, named for their characteristic “tree-like” morphology [3]. LCs are classically considered the tissue-resident macrophages of the skin [4,5]. In healthy skin, these MHC class II-expressing APCs [6,7] reside primarily in the suprabasal epidermis, a depth to which UV penetrates easily. UV radiation has been shown to influence several aspects of LC networks and function. Much of our understanding of the immunosuppressive effects of UV radiation has been elucidated through murine models of contact hypersensitivity (CHS), which is a delayed type of cell-mediated immune response.

Epidermal LCs are essential mediators in the development of CHS responses [8]. The extent of CHS produced in response to a contact allergen correlates with the density of local LCs in the epidermis at the site of initial sensitization. This was first shown in an experiment by Toews et al., in which initial sensitization to the contact allergen 2,4-dinitrofluorobenzene (DNFB) on the abdominal skin, which naturally has a higher density of LCs, triggered a stronger hypersensitivity reaction upon rechallenge compared to sensitization on tail skin, where LCs are relatively scant [8]. Irradiation of the skin with a short course of 290–320 nm UV radiation (UVB) prior to sensitization significantly attenuated the capacity to subsequently develop a CHS response to DNFB. Furthermore, UV-irradiated mice were unable to mount an immune response to re-sensitization to DNFB applied to a distant site at later time points as would be expected in UV-naïve mice, indicating the induction of specific immune tolerance [8]. Later studies by Cruz et al. found that injection of isolated Ia^+^ LCs that had been irradiated and conjugated to hapten ex vivo into normal, wildtype mice resulted in a diminished ability to mount a CHS response, and the development of long-lasting immune tolerance compared to mice injected with non-UV-irradiated LCs [9].

The specific cellular and molecular effects on LCs attributed to UV radiation can be highly variable, based on the dose and range of UV employed and the timing of sampling post-irradiation. Toews et al. found that LCs, as identified by ATPase-positive staining, are transiently depleted at the site of local UVB irradiation. The few ATPase^+^ cells remaining following irradiation exhibit altered morphology [8]. Exposure to high doses of UVB ranging from 400 to 4000 J/m^2^ causes LCs at the site of irradiation to become rounded and swollen, with a reduction in dendritic processes [1,8,10]. Other studies have shown that irradiation with lower doses of UVB 120–2000 J/m^2^ results in LCs with elongated dendritic processes [2,11]. Experiments in murine and human skin by Aberer et al. suggest that the apparent loss of LCs may be reflective of a loss of surface markers (e.g., Ia antigens), rather than a true depletion of LCs [12]. Further immunohistochemistry and electron microscopy has suggested that DCs experience both a change in surface marker expression and cell damage or death in response to UV irradiation [2].

As molecular techniques improve and the classification of DC populations becomes increasingly nuanced, it has become clear that not only are the effects of UV radiation context-dependent, but various cell subpopulations also are impacted in unique ways [13]. Nakagawa et al. have shown that UVB radiation has distinct effects on different LC subpopulations, defined by relative HLA-DR expression [14]. In response to UVB exposure, the relative number of viable HLA-DR^HI^ LCs decreases over time, but this subpopulation exhibits evidence of maturation (downregulation of CD1a; upregulation of CD80, CD86, CD54, CD40, CD83) compared to unexposed controls. UVB potentiates the activity of viable HLA-DR^HI^ LCs through augmented expression of costimulatory molecules, such as TNF-α and proinflammatory cytokines. HLA-DR^low^ LCs, on the other hand, fail to mature in response to UVB, and exhibit robust annexin V binding, indicating apoptosis [14].

While epidermal LCs have been studied extensively, a handful of studies have examined the importance of dermal DCs in UV-induced immunosuppression. Dermal langerin^+^CD103^+^ cells, which constitute two-thirds of langerin^+^ cells in the skin-draining lymph nodes, are derived from the bone marrow, and are constitutively expressed in the dermis [15,16,17]. Conditional deletion of this population of DCs via radiation reduces the UV-induced suppression of CHS and the suppression of CD8^+^ T cell responses to epicutaneous immunization to OVA [18], suggesting that epidermal langerin^+^ cells are dispensable in the induction of UVB-induced immunosuppression [18]. However, experiments utilizing transgenic mice expressing diphtheria toxin receptors on langerin^+^ cells have suggested that epidermal langerin^+^ cells are required for UVB-induced immune suppression. In these studies, when administered 10 days following diphtheria toxin-mediated depletion of langerin^+^ cells, at which point dermal langerin^+^ cells, but not epidermal langerin^+^ cells, had repopulated, UVB was unable to effectively suppress CHS [19].

### 2.2. Conventional Dendritic Cells (cDCs)

cDCs that express CD11c and MHC class II antigens have been characterized in mice and humans [20]. Based on their phenotype, they are generally classified into cDC1 and cDC2. In addition to CD11c and MHC class II antigens, murine cDC1 cells express XCR1, IRF8, and CD103, whereas cDC2 cells express CD11b and IRF4. In addition to CD11c and MHC class II antigens, human cDC1 cells express CD304 (BDCA3), CD141 (BDCA4), and XCR1, whereas cDC2 cells express CD1c (BDCA1) and CD11b. cDCs can be detected in the skin, blood, and lymphoid tissues. Many studies investigate the roles of CD11c^+^ DC in UV-induced immune suppression in mice and humans. However, less is known about the specific roles of cDC1 and cDC2 cells in the process.

CD11c^+^ DCs have been implicated in the systemic immunomodulatory effects of UV exposure. In addition to influencing LCs locally at the site of irradiation, UV radiation of the skin leads to functional alterations in distal bone marrow-derived DCs. Cultured bone marrow cells from UV-irradiated BALB/c mice have been shown to produce greater levels of IL-10 and prostaglandin E2 (PGE2), relative to bone marrow cells isolated from non-irradiated controls [21]. When ex vivo bone marrow cell cultures are stimulated with IL-4 and granulocyte macrophage colony stimulating factor (GM-CSF) ex vivo, CD11c^+^ cells are generated. In an adoptive transfer experiment, Ng et al. found that the transfer of bone-marrow-derived CD11c^+^ cells from UV-irradiated mice into naïve recipient mice mitigates CHS responses and confers long-lasting suppression of memory responses to a contact allergen [21]. This mechanism of UV-induced systemic immune suppression was found to be mediated by PGE2, as the effects were blocked by pretreatment with indomethacin [21]. Additional studies have similarly suggested that long-lasting immunosuppressive effects may be epigenetically imprinted in hematopoietic stem cells [22,23,24].

CD11c^+^ DCs in skin-draining lymph nodes play an important role in UV-mediated suppression of CHS responses. This is thought to be due to their role in IL-12 production. IL-12 has been shown to protect against UV-induced CHS suppression [25,26,27]. Augmenting IL-12 secretion by CD11c^+^ cells by draining lymph nodes with the Toll-like receptor 7 (TLR7)-agonist Imiquimod prevents UV-induced suppression of hapten sensitization and CHS [28].

Locally, exposure to 2MED of 312 nm narrow-band UVB leads to a significant increase in the number of CD11c^+^ DCs in the dermis of individuals with Fitzpatrick II-III skin [29]. More specifically, the CD11c^+^ DCs seen 24 h following irradiation include immunosuppressive CD11c^+^BDCA3^+^ subsets that have been shown to exert immunosuppressive effects through IL-10 and induction of regulatory T cells [30], as well as immature, inflammatory CD11c^+^BDCA1^−^ BDCA3^−^ subsets that poorly co-localize with dendritic cell lysosomal-associated membrane glycoprotein (DC-LAMP) on immunofluorescence imaging, and express tumor necrosis factor (TNF)-α and TNF-related apoptosis-inducing ligands (TRAILs) [29].

### 2.3. CD11b^+^ Myeloid Cells

Amongst the inflammatory milieu seen in the skin following UV irradiation are CD11b^+^ myeloid cells [31]. CD11b^+^ myeloid cells can be distinguished from LCs of the skin by the absence of surface CD1a^+^. UV-induced CD36^+^CD11b^+^CD1a^−^ cells are robust producers of IL-10 mRNA and protein [32]. Conversely, UV-exposed keratinocytes and CD1a^+^ LCs express little to no IL-10 mRNA [32]. Further studies show that a ligand of CD11b, iC3b (a complement component C3), is deposited in UV-exposed skin, and is localized in apposition to infiltrating CD11b^+^ myeloid cells. Stimulating CD11b^+^ cells with iC3b significantly induces IL-10 mRNA and protein. In contrast, it suppresses IL-12 mRNA and protein [33]. Mouse studies demonstrate that the depletion of CD11b^+^ cells prevents UV-induced immune suppression [34]. In contrast, the activation of CD11b^+^ cells by complement component C3 is required for UVB-induced CD11b^+^ cell migration into the skin and immune suppression [35]. It is to be noted that cDC2 cells express CD11b^+^ [20]. However, it remains to be determined whether cDC2 cells play a role in UV-induced immune suppression.

## 3. Mechanisms for UV-Induced Regulation of DC Activity

**Figure 1:**
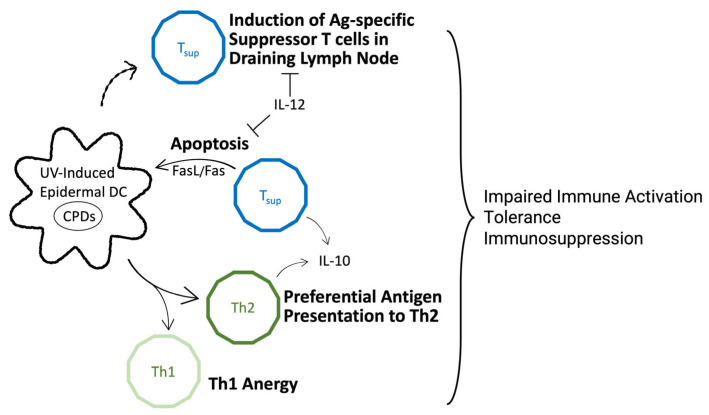
Graphical summary of immunosuppressive actions of UV-induced dendritic cells and T cell populations. Ag = antigen, CPDs = cyclobutene pyrimidine dimers, DC = dendritic cell, Th = T helper cell, Tsup = suppressor T cell.

UV radiation suppresses the immune system by modulating DC function in two main ways: it impairs their antigen-presenting ability, and it promotes tolerance by inducing regulatory, immunosuppressive T cell populations. Together, these effects block immune activation and enhance suppression, resulting in a net immunosuppressive outcome (Figure 1).

### 3.1. Inactivation of DCs

UV-induced DNA damage, particularly the generation of cyclobutane pyrimidine dimers (CPDs), is partially responsible for the impairment of APC function in DCs. Studies have demonstrated that treating UV-irradiated skin with liposomes containing DNA excision repair enzymes, such as T4 endonuclease V, reduces the number of CPD-containing DCs in the draining lymph nodes and restores APC function [36]. In vitro, repairing CPDs using liposomes containing photolyase (a light-activated DNA repair enzyme) also restores the APC function of DCs from UV-irradiated murine skin [37].

UV radiation also disrupts antigen presentation through the induction of DC apoptosis. While controlled apoptosis is essential under normal conditions for maintaining immune homeostasis and self-tolerance [38], UV radiation interferes with this balance. UV radiation prevents the maturation of, and induces apoptotic cell death in, a specific subpopulation of LCs characterized by low HLA-DR expression [14]. Gene knockout mice deficient in pro-apoptotic BH3-interacting death domain protein (Bid) exhibited significant resistance to UV-induced LC depletion, suppression of local CHS responses, and tolerance to haptens. Notably, these mice also displayed reduced CPD accumulation in lymph nodes following UV exposure, compared to wildtype control mice [39]. Additionally, co-culture experiments have demonstrated that hapten-pulsed DCs underwent Fas/FasL-mediated apoptosis when exposed to T cells from UV-irradiated mice. DCs from Lpr and gld mice, which lacked functional *Fas* and *FasL* genes, were resistant to apoptosis induced by UV-induced T suppressor cells. Notably, interleukin-12 (IL-12) can rescue DCs from Fas/FasL-mediated apoptosis, offering a potential therapeutic pathway [27]. Moreover, apoptotic DCs express CD200 (OX-2), a target of p53, which attenuates proinflammatory cytokine production in response to self-antigens in vitro. In vivo, CD200 is essential for UVB-mediated tolerance to self-antigens, as its absence disrupts this immunosuppressive pathway [40]. CD200R is expressed on myeloid-derived APCs and some T cells, facilitating the immunoregulatory effects mediated by CD200/CD200R signaling [41,42].

### 3.2. Tolerogenic DCs

Tolerogenic DCs are specialized immunosuppressive APCs that promote immune tolerance, or non-reactivity, to specific antigens. These effects are primarily mediated through interactions with T lymphocytes and the release of anti-inflammatory cytokines. By modulating T cell activity, tolerogenic DCs can induce T cell anergy, promote T cell apoptosis, and facilitate the differentiation of T regulatory (Treg) cells [43].

The activity and function of tolerogenic DCs are not lineage-specific, but are highly influenced by their surrounding microenvironment [43,44]. Notably, UV radiation, particularly UVB, can convert LCs from immunologically active APCs into tolerogenic ones [19]. UV-induced Treg cells are antigen-specific [45]. Treg cells in UV-irradiated skin have been shown to release immunosuppressive IL-10 [46] and express lymph node-homing receptors such as CD62L, while simultaneously lacking skin-homing molecules such as E-selectin or P-selectin [47]. Similarly to naturally occurring, thymic Tregs, UV-induced Tregs express CD25 following hapten sensitization [47]. UV-induced Treg cells also express cytotoxic T-lymphocyte antigen-4 (CTLA-4), which is known to negatively regulate T lymphocyte function [48]. Depleting CLTA-4^+^ cells or blocking CTLA-4 signaling with a monoclonal antibody blocks the transfer of UV-induced immune suppression in adoptive transfer experiments [48,49].

### 3.3. DCs and the Development of T Lymphocyte Populations in the Context of UV-Induced Immune Suppression

#### 3.3.1. CD4^+^ Treg Cells

The generation of Tregs following UV exposure involves the migration of LCs exhibiting DNA damage into draining lymph nodes [50]. In experiments in which UV-mediated DNA damage was mitigated by IL-12, the generation of UV-induced Tregs was prevented [25,26,51,52]. In vitro experiments examining DC–Treg interactions in isolation from the influence of surrounding cell types have confirmed that the repair of CPDs in UV-irradiated DCs prevents the induction of suppressor T cells [37]. Unlike the UV-induced suppression of T cells, the induction of Tregs specifically requires the presence of damaged, yet viable, UV-irradiated LCs in the lymph nodes. This distinction is further supported by experiments demonstrating that killing LCs (as opposed to damaging them) with the steroid mometasone inhibits sensitization to contact allergens, but does not lead to Treg production [19].

#### 3.3.2. CD4^+^ T Helper Cells

Under standard biologic conditions, DCs interact with naïve CD4^+^ T cells to promote their differentiation into either type 1 (Th1), type 2 (Th2), or type 17 (Th17) helper cells [53,54]. Th1 cells are generally considered to be inflammatory, as they secrete IFN-γ and aid B lymphocytes in the production of complement-fixing antibodies. The primary effector cytokine of Th2 cells is IL-4, and these cells are associated with allergic, IgE-mediated reactions. Anti-inflammatory IL-10 is also a Th2-type cytokine [55]. UV exposure stimulates the production of Th2 cytokines in the skin-draining lymph nodes more so than Th1 cytokines [56,57]. LCs develop a skewed propensity for presenting to Th2 cells over Th1 cells following UV irradiation [58]. In the setting of *Borrelia burgdorferi* infection, UV irradiation results in diminished levels IgG2a and IgG2b, which typically require functional Th1 activity, and in increased levels of IgG1 antibody, which is supported by Th2 function [59]. Additionally, antigen presentation by UV-irradiated DCs leads to Th1 anergy and tolerance to restimulation with normal antigen-bearing DCs [60]. Th17 cells primarily produce IL-17, which has a wide spectrum of effects on innate and acquired immunity [61,62]. IL-17 is an important inflammatory cytokine for CHS [63]. A deficiency in IL-17-mediated immune responses will suppress CHS [64,65]. UV radiation increases IL-17 production in the skin [66]. Evidence indicates that the knockout of the IL-17 receptor A gene diminishes UVB-induced immune suppression [67]. The mechanisms for such processes are attributed to reduced UVB-induced CD11b^+^ myeloid cells and Treg cells in the draining lymph nodes. Further studies are required to elucidate these mechanisms.

## 4. The Role of DCs in UV-Induced Skin Diseases

The dysregulation of immune responses due to UV radiation has profound implications for various skin diseases, three of which will be highlighted here.

### 4.1. Cutaneous Lupus Erythematous (CLE)

CLE is a subset of lupus erythematous that is characterized by its effects on the skin. CLE is classified into three subtypes: acute, subacute, and chronic. However, clinically, considerable overlap exists amongst these subtypes, often making differentiation challenging [68,69]. CLE may develop as a standalone condition or as a skin-related manifestation of systemic lupus erythematosus [70,71]. UV radiation plays a multifaceted and pleiotropic role in CLE pathogenesis, involving induction of keratinocyte damage, apoptosis, and necrosis. These processes are mechanistically driven by the generation of reactive oxygen species, DNA modifications, and autoantigen expression, which ultimately activate immune pathways—particularly the type I interferon (IFN) system, via cyclic GMP-AMP synthase-stimulator of IFN genes (cGAS-STING) and IFNκ—leading to immune cell recruitment, autoreactivity, and disease flares (reviewed by Patel et al.) [71].

With specific regard to UV-induced DCs, UV exposure induces the accumulation of plasmacytoid DCs (pDCs) via TLR signaling [72,73]. These pDCs are major producers of type I IFNs, which are key mediators in lupus erythematous [72,73]. The number of pDCs in CLE lesions correlates with the degree of immune cell infiltration, highlighting their contribution to disease progression [74]. pDCs in CLE are commonly seen in close association with mature dermal CD208^+^ DCs or with cytotoxic CD8^+^ T cells along areas of damage in the dermal–epithelial junction [74]. Mechanistically, pDCs endocytose immune complexes, and the activation of TLR7 and TLR9 leads to type I IFN production [75,76,77]. In vitro experiments suggest that this process is mediated by the Fc receptor CD32 [78,79]. The humanized IgG1 monoclonal antibody BIIB059 targets blood DC antigen 2 (BDCA2) on the surface of pDCs, effectively downregulating IFN production [80]. In a phase 2 clinical trial, BIIB059 demonstrated significant improvements in clinical endpoints for CLE [81,82,83]. Other mechanisms, such as the activation of macrophages and cDCs by keratinocyte debris or cell death, have also been proposed as contributors to IFN production [84], further underscoring the complexity of CLE pathophysiology and the interplay between UV exposure and immune activation.

### 4.2. Polymorphic Light Eruption (PLE)

PLE is a common photodermatitis, characterized by pruritic skin lesions, most often papulovesicular in nature, that develop hours to days after sunlight exposure [85]. The prevalence of PLE has been shown to correlate with geographic latitude, with higher rates observed in regions farther from the equator [86]. The condition typically manifests within the first three decades of life, and is more commonly reported in females [87,88]. While the lesions typically arise early in the sunny season, repeated UV exposure often leads to a photohardening effect, reducing the likelihood of recurrence as summer progresses. This phenomenon can also be induced through preventive phototherapy [89].

The pathophysiology of PLE remains incompletely understood, but it is believed that patients exhibit resistance to UV-induced immunosuppression, fostering a microenvironment that is conducive to aberrant immune responses to photoinduced stimuli. Specifically, LCs in patients with PLE demonstrate impaired UV-induced mobilization, a defect associated with diminished neutrophil infiltration and reduced expression of key cytokines, such as TNF-α, IL-4, and IL-10, following UV exposure [90,91,92]. This resistance to tolerogenic signaling likely disrupts immune homeostasis, contributing to the pathological skin responses that are characteristic of PLE.

### 4.3. Skin Cancer

The role of UV-induced DCs, particularly LCs, in skin cancer pathogenesis is both complex and critical [13]. LCs, under normal circumstances, serve as APCs. UV radiation alters this dynamic by inactivating or modifying LCs, impairing their ability to mount an effective immune response, as discussed above. As posited by Toews et al., if LCs primarily function to present antigens and prevent immunologic tolerance to antigens, their chronic inactivation by UV radiation permits neoantigens from malignantly transformed cells to be perceived as tolerogens [8]. This shift from immune surveillance to tolerance facilitates the unchecked growth of malignant cells, setting the stage for skin cancer development.

Experimental evidence supports this pivotal role of UV-induced changes in LCs in skin cancer pathogenesis. Studies by Kripke and colleagues have demonstrated that UV-irradiated mice fail to reject highly antigenic UV-induced tumors, which would otherwise be eliminated by normal syngeneic recipients [93,94]. This inability to reject tumors correlates with a UV-induced decrease in epidermal LCs, which in turn diminishes antigen-presenting activity in skin-draining lymph nodes. The reduction in DCs within the skin disrupts the immune response, and highlights the importance of LCs in initiating tumor-specific immunity [95].

Moreover, the interaction between LCs and UV-induced carcinogenesis is nuanced. Despite their protective role under normal conditions, LCs may paradoxically augment UV-induced cutaneous carcinogenesis. Studies indicate that epidermal tissues with intact LC networks develop UV-induced tumors more readily than epidermal tissues with scant LCs, independently of CPD formation following UV exposure [96]. This paradox may arise from LCs promoting immune tolerance or fostering an environment that is conducive to tumor growth. Mutant p53 islands, often associated with proliferating mutant keratinocytes, are frequently found in close proximity to LCs, further implicating these cells in tumor progression. Therefore, while LCs play a critical role in immune surveillance, their UV-induced alterations highlight their dual role in the pathogenesis of skin cancer.

## 5. Conclusions

To conclude, UV radiation profoundly impacts DC function, inducing a tolerogenic state that suppresses immune activation and promotes immune tolerance. This immunomodulatory effect is mediated by complex cellular and molecular pathways, including alterations in LCs, cDCs, and CD11b^+^ myeloid cells. UV exposure impairs antigen presentation, induces apoptosis, and drives the generation of regulatory T cells, culminating in the promotion of suppressive immune microenvironments and reactions at both a cutaneous and systemic level.

Insights from studying UV-induced tolerogenic DCs deepen our understanding of the mechanisms underlying immunosuppression, and highlight their clinical relevance in the pathogenesis of autoimmune skin disorders and skin cancers. Significant progress has been made in identifying these tolerogenic DCs and exploring their potential therapeutic implications.

## 6. Perspective

Our knowledge of the underlying mechanisms of UV-induced immunosuppression remains incomplete. Future research leveraging advanced molecular techniques and single-cell analyses will be crucial for refining our understanding of UV-induced immunosuppression. These approaches will enable the detailed characterization of specific tolerogenic DC subsets, and elucidate the precise pathways by which UV radiation influences DC function and by which UV-induced tolerogenic DCs induce Treg cells and immune tolerance. Furthermore, such advancements will enable innovative strategies in mitigating the adverse effects of UV radiation and harnessing tolerogenic DCs for immunomodulation in therapeutic contexts.

## Figures and Tables

**Table 1:** Common cellular markers and methods of identification used in the study of UV-induced dendritic cell populations referenced in this review.

	Identification Method	Special Considerations
LCs	- Markers for histologic and immunohistochemical examination: - ATPase activity - Ia^k^ and Ia^d^ - CD1a - Langerin (CD207) - CD103^−^ epidermal LCs vs. CD103^+^ dermal LCs - Electron microscopy of ultrastructural appearance and presence of Birbeck granules	- UV exposure attenuates ATPase staining more so than CD1a and Ia expression [1] - LCs are distinguished from dendritic melanocytes by a lack of cytoplasmic pigment granules [2] - UV exposure alters LC morphology
cDCs	- Markers for histologic and immunohistochemical examination: - CD11c^+^ CD123^−^ - CD11c^+^ BDCA3^+^ immunosuppressive subset - CD11c^+^ BDCA1^−^ immature, inflammatory	- UV induces changes to CD11c^+^ populations in the skin, draining lymph nodes and bone marrow - Commonly assessed via flow cytometry
CD11b^+^	- Histologic examination or flow cytometry for the following markers: - CD11b^+^CD36^+^CD1a^−^ - Gr-1 (Ly-6G/Ly-6C) - Human: CD11b^+^ CD1a^−^HLA-DR^+^	- Commonly assessed via flow cytometry

cDC = conventional dendritic cell, LC = Langerhans cell, UV = ultraviolet, DC = dendritic cell.

## Data Availability

No new data were created or analyzed in this study.

## Abbreviations

The following abbreviations are used in this manuscript:

APC	Antigen-presenting cell
BDCA	Blood DC antigen
Bid	BH3-interacting death domain protein
cDC	Conventional dendritic cell
CPD	Cyclobutene pyrimidine dimer
CHS	Contact hypersensitivity
CLE	Cutaneous lupus erythematous
DC	Dendritic cell
DNFB	2,4-dinitrofluorobenzene
IFN	Interferon
IL	Interleukin
LC	Langerhans cell
MHC	Major histocompatibility complex
pDC	Plasmacytoid dendritic cell
Th1	T helper 1
Th2	T helper 2
Th17	T helper 17
Treg	Regulatory T cell
UV	Ultraviolet
UVB	Ultraviolet B
